# Architecture of viral replication factories

**DOI:** 10.18632/oncotarget.5900

**Published:** 2015-09-28

**Authors:** Yongliang Zhang, Xiuling Cao, Dawei Li

**Affiliations:** State Key Laboratory of Agro-Biotechnology and Ministry of Agriculture Key Laboratory of Soil Microbiology, College of Biological Sciences, China Agricultural University, Beijing, People's Republic of China

**Keywords:** architecture, virus replication complexes

Compartmentalization of intracellular membrane is a marked characteristic during the pathogenesis of many viruses, which provides a microenvironment for virus to evade the host RNA interference defense response on one hand, and confines various host factors as well as metabolites in a stable space so that they can act in concert to complete virus replication cycle [1]. Recently, advanced imaging technologies such as the three-dimensional (3D) electron tomography have increasingly become a robust tool for further understanding how the cells were controlled to form the virus factory [2].

3D analysis of various virus replication complexes (VRCs) revealed that, albeit with their own distinct traits, structural similarities are present among different VRCs, suggesting viruses might adopt conserved strategies to build the replication factory during long-term evolution. For example, Kopek *et al*., for the first time, resolved the 3D architecture of VRCs, which shows the outer mitochondrial membrane-invaginated 50 nm vesicles (spherules) in the flock house virus (FHV)-infected *Drosophila* cells [3]. Recently, we also generated the first 3D architecture of VRCs in virus-infected plant cells [4]. Typical membrane spherules derived from the endoplasmic reticulum could also be observed despite these two viruses were evolutionarily far removed from each other and taxonomically unrelated. In fact, similar shape of membrane-invaginated vesicles was also reported in other organelles such as peroxisome, chloroplast and vacuole during virus infection [1]. The morphological convergence of VRCs in diverse organelles under different viral infections implies that two issues, at least, warrant further investigation. 1) Common molecular mechanisms may exist in the process of forming membrane-associated spherules. One obvious feature is the curvature of the membrane. Recently, a class of membrane proteins named reticulons, which is essential for stabilizing highly curved ER membrane tubules, had been proved to play important roles in viral RNA replication compartment formation [5]. This is a good startpoint, yet more research is needed to elucidate how the virus manipulate the cell to initiate the membrane wrapping and how the host cellular factors were hijacked to maintain such unstable membrane status. Meanwhile, another phenomenon deserves our attention is that membrane curvature leads to surface area expansion, resulting in increased chances of contacting with the outer cytoplasm. Recent studies indicated that the contact sites between the membranes of different organelles play an important role in favoring the exchange of metabolites and information, particular the ER that involved in most membrane contact sites (MCSs) within the cell [6]. It is interesting to study the function of these MCSs in mediating the translocation of host molecules under the stress of viral infections. 2) The establishment of almost all the VRCs inevitably involved the participation of lipid molecules, and accumulating evidence substantiates the critical roles of membrane lipid played in the virus replication cycle. Kai *et al* demonstrated that the enrichment of phosphatidylethanolamine at the membrane-bound VRCs is an essential step for a (+)RNA virus replication [7]. Thus, exploring the interaction of viruses with the lipid biosynthetic and transport machinery is also an intriguing subject in the future study.

It should also be pointed out that one or several virus-encoded proteins usually act as key triggers of VRCs morphogenesis. Sometimes expression of a single viral protein alone in the cells could form identical cytopathological structures with that of corresponding viral infections [4]. Hence, using these viral proteins as a probe to screen the host factors or identify cellular signaling pathways contributing to the formation of VRCs will continue to be a routine but significant work in the subsequent research.

Taken together, the 3D views of viral replication factories provide new insight into virus-host interaction and should greatly facilitate the discovery of potential targets for antiviral therapeutic intervention.

## References

[1] Verchot J (2011). Curr. Opin. Virol.

[2] Risco C. (2014). Annu. Rev. Virol.

[3] Kopek B.G. (2007). PLoS Biol.

[4] Cao X. (2015). J.

[5] Diaz A. (2010). Proc. Natl. Acad. Sci. U.S.A.

[6] Helle S.C.J. (2013). Biochim.

[7] Xu K. (2015). Proc. Natl. Acad. Sci. U.S.A.

